# The predictive value of early acute kidney injury for long-term survival and quality of life of critically ill patients

**DOI:** 10.1186/s13054-016-1416-0

**Published:** 2016-08-03

**Authors:** Ivo W. Soliman, Jos F. Frencken, Linda M. Peelen, Arjen J. C. Slooter, Olaf L. Cremer, Johannes J. van Delden, Diederik van Dijk, Dylan W. de Lange

**Affiliations:** 1 Department of Intensive Care Medicine, University Medical Center Utrecht, Heidelberglaan 100, Utrecht, 3584 CX The Netherlands; 2 Department of Epidemiology, Julius Center for Health Sciences and Primary Care, Universiteitsweg 100, Utrecht, 3584 CG The Netherlands; 3 Department of Medical Humanities, Julius Center for Health Sciences and Primary Care, Universiteitsweg 100, Utrecht, 3584 CG The Netherlands

**Keywords:** Acute kidney injury, AKI, HRQoL, Health related quality of life, Intensive care, Critical care, Outcome, Long-term, Prognosis

## Abstract

**Background:**

Prognostic factors for the combination of long-term survival and health-related quality of life (HRQoL) after intensive care unit (ICU) stay have not yet been studied. Our aim was to assess whether early acute kidney injury (eAKI), AKI occurring on the first day of ICU admission, is an independent predictor of this combined one-year outcome.

**Methods:**

We included all patients admitted to the mixed ICU of the University Medical Centre Utrecht between July 2009 and April 2013, excluding patients with chronic dialysis, cardiac surgery, and length of stay shorter than 24 hours. eAKI was defined using the risk, injury, failure, loss, end-stage renal failure (RIFLE) classification, using a newly developed algorithm to classify AKI based on routinely collected patient data. In one-year survivors, HRQoL was measured using the EuroQoL 5D-3L™ (EQ-5D) questionnaire. The primary outcome measure was “poor outcome”, defined as an EQ-5D index score <0.4 or death after one year follow up. A multivariable Poisson regression model was performed to adjust for age, comorbidities, admission type and severity of disease factors.

**Results:**

We enrolled 2,420 patients, of whom 871 (36.0 %) died within one year. An additional 286 of 1549 one-year survivors (11.8 %) experienced low HRQoL. The respective incidence of the RIFLE classes, risk, injury and failure, were 456 (18.8 %), 253 (10.5 %) and 123 (5.1 %). After adjustment for other covariates, the RIFLE classes, injury and failure, were independently associated with poor outcome (adjusted relative risk 1.14, 95 % CI 1.01, 1.29; *p* = 0.03, and 1.25, 95 % CI 1.01, 1.55; *p* = 0.04), when compared to no eAKI patients . The constituents of this composite outcome were also analysed separately. In a Cox regression model the RIFLE classes, injury and failure, were significantly associated with mortality (adjusted hazard ratio 1.35, 95 % CI 1.11, 1.65; *p* <0.01, and 1.78, 95 % CI 1.38, 2.30; *p* <0.01). In one-year survivors specifically, none of the RIFLE classes were significantly associated with low HRQoL.

**Conclusions:**

ICU patients with moderate or severe AKI during the first 24 hours have a higher probability of mortality or low HRQoL (combined poor outcome), one year after ICU admission. Together with other available early prognostic factors, information on early acute kidney injury could improve informed decision-making on the continuation or withdrawal of treatment in ICU patients.

**Electronic supplementary material:**

The online version of this article (doi:10.1186/s13054-016-1416-0) contains supplementary material, which is available to authorized users.

## Background

Early during ICU admission clinicians often find it difficult to predict the long-term outcome of critically ill patients [1]. Even during the course of an intensive care unit (ICU) admission the prognosis may remain unclear. In order to support decision-making on the continuation or withdrawal of ICU treatment, identifying valid clinical predictors early during ICU admission is particularly relevant [2].

The first day of ICU admission is critical for prognosis. Its clinical relevance is made clear by the high prognostic value of disease severity scores based on the first day of ICU admission. A multitude of physiologic variables are included in models such as the Acute Physiology and Chronic Health Evaluation (APACHE) and Simplified Acute Physiology Score (SAPS) [3, 4]. However, only the presence of specific ICU complications such as acute kidney injury (AKI) is taken into account. Worldwide established and detailed classification systems for complications identifying different levels of severity, were not used.  A considerable number of critically ill patients develop AKI during their ICU stay [5]. Over twenty percent of all general ICU patients do so within 24 hours of ICU admission [6]. Moreover, when AKI does accompany critical illness, it is a risk factor for increased mortality, up until one year after ICU admission [5–14]. Early AKI (eAKI) could be an especially prevalent warning sign of poor long-term outcomes. If so, it could be used as a building block for personalized prognoses.

Multiple studies have investigated predictors and models for the short-term prognosis of the critically ill. As a consequence, scoring systems such as APACHE IV and SAPS 3 have been developed for risk stratification [3, 4]. These studies focussed on associating predictors and models with hospital mortality. To facilitate decision-making on continuing or withdrawing treatment in the ICU, however, patients and their relatives usually want to be informed about the chances of survival beyond hospital discharge. Often, they want to take the expected quality of life into account. Health-related quality of life (HRQoL) has only been studied scarcely as the outcome in prognostic factor studies [15–17]. Especially in the general ICU population studies the investigation of predictors of HRQoL is rare. Furthermore, prognostic factors of a combination of survival and HRQoL have not yet been studied.

Therefore, the aim of this study was to investigate whether the occurrence and severity of eAKI, which is defined as AKI occurring during the first 24 hours of admission, in a mixed ICU population is independently associated with one-year mortality and HRQoL.

## Methods

### Study design, setting and participants

All patients admitted consecutively to the mixed ICU of the University Medical Center Utrecht from July 2009 to April 2013, without chronic dialysis prior to ICU admission, were eligible for inclusion. In patients with multiple ICU admissions within this period, only the first ICU admission was used in the analyses. Patients under 16 years of age and those with a length of stay shorter than 24 hours were excluded. Patients admitted to the ICU after cardiac surgery were excluded because of the low incidence of AKI [18], and low risk of poor outcome in these patients in general [16]. The Institutional Review Board (IRB) of the University Medical Center Utrecht approved the study protocol and waived the need for informed consent when working with anonymised patient and follow-up data (UMC Utrecht IRB protocol number 10/006).

### Data collection and follow up

The following data were collected: serum creatinine levels, urine output per hour within the first 24 hours, age, sex, pre-ICU hospital length of stay, admission type, Charlson Comorbidity Index [19, 20], the need for mechanical ventilation, confirmed infection and the acute physiology score (APS, as part of the APACHE II score) within the first 24 hours of admission. These variables were prospectively collected according to strict definitions, as part of a national registry used for benchmarking [7]. Data on the Charlson Comorbidity Index were obtained from the electronic patient files, as was described previously [21].

After hospital discharge, patient survival was tracked using the municipal registry. All patients surviving one year after ICU admission were sent the EuroQoL 5D-3L™ (EQ-5D) HRQoL questionnaire [22]. If this questionnaire had not been returned within six weeks, the questionnaire was resent and patients were reminded by telephone to return the questionnaire. More details on the definitions of the Charlson Comorbidity Index and the EQ-5D can be found in Additional file 1.

### Early AKI

The presence of eAKI was determined according to the risk, injury, failure, loss, end-stage renal failure (RIFLE) classification [5]. This classification is based on measurements of serum creatinine, urine output per hour and the use of renal replacement therapy. A risk; injury; failure (RIF) classification of renal impairment (analogous with the RIFLE system) was used. An algorithm was developed to determine the presence of these acute RIFLE classes within the first 24 hours of ICU stay, based on routinely collected data. RIFLE based on serum creatinine was scored by calculating the factor of change in serum creatinine from baseline. The baseline was defined as the lowest serum creatinine value in the 6 months prior to ICU admission, when available in the hospital laboratory registry. If this was unavailable, the lowest serum creatinine during the first day of admission was used as baseline. RIFLE based on urine output was scored per hour, where 6, 12 or 24 hour stretches of oliguria or anuria were scored according to the RIFLE classification. Hours with missing urine output were replaced by dividing the first known urine output over the stretch of missing hours. This was done for missing periods up to 6 hours, including the hour with the known urine output measurement. Renal replacement therapy was scored based on parameters indicating a running dialysis and invasive therapy registration. The highest (i.e., worst) acute RIFLE class based on serum creatinine or urine output attained during the first 24 hours of admission was used to classify eAKI in included subjects. Subjects with renal replacement therapy were scored as “failure” regardless of urine or serum creatinine. More details on this algorithm can be found in Additional file 2.

### Outcomes

The primary outcome measure was one-year “poor outcome”. This outcome was defined as a composite of death or low HRQoL at one year follow up. To study the contribution to the composite endpoint, one-year survival, and HRQoL in the one-year survivors were analyzed as separate secondary outcomes.

HRQoL was measured using the EQ-5D. This questionnaire consists of five questions each representing a dimension of HRQoL (mobility, self-care, usual activities, pain or discomfort, anxiety or depression). Patients assigned a score of no, little or many problems to each of these dimensions. The results were indexed on a scale between 1 (full health) and 0 (dead) according to the weighting scheme for the Dutch population [22].

We defined low HRQoL as an EQ-5D index of 0.4 or below. Patients who qualified their health state as such are on par with those with moderate to severe amyotrophic lateral sclerosis (mean EQ-5D index 0.56–0.27) [23], patients suffering from dementia with depression (mean EQ-5D index 0.37) [24], or patients with a severe to extreme depressive episode (median EQ-5D index 0.57–0.29) [25].

### Additional predictors of outcome

Based on the constituents of most benchmark prediction models (e.g., APACHE and SAPS) additional predictors were selected. These were age, gender, pre-ICU hospital length of stay, admission type (medical/elective surgical/urgent surgical), Charlson Comorbidity Index, the need for mechanical ventilation, confirmed infection and the APACHE II acute physiology score in the first 24 hours of admission [26] (excluding the points for creatinine). The association between eAKI and outcomes was adjusted for overlapping information on the predictive value of these eight additional predictors.

### Missing data

Missing EQ-5D data were expected to occur in non-responding one-year survivors. Multiple imputation was used to replace the missing EQ-5D dimension scores of non-responding survivors [27–30]. A total of 35 imputation datasets were created. Further details can be found in Additional file 1.

### Data analysis

Baseline-characteristics and outcomes were compared across RIFLE classes using the Chi^2^ test for categorical variables and were compared non-parametrically using the Kruskal-Wallis test for continuous variables. The association between RIFLE and poor outcome was analysed using multivariable Poisson regression analysis, modified for binomial outcomes, adjusting for the aforementioned additional risk factors [31]. To adjust for any non-linearity in the association of continuous variables with outcome, fractional polynomial transformations were added to the model [32]. Cox proportional hazard regression analysis was conducted to assess the association between eAKI and one-year survival. Any violation of the proportional hazard assumption was verified and where necessary adjusted for by including an interaction term with time in the regression model. In one-year survivors we investigated the association between eAKI and low HRQoL by using a similar multivariable modified Poisson regression analysis as for the primary outcome. Rubin’s rule for pooling multiple imputation datasets was used to arrive at correct effect estimates and standard errors [27].

All statistical analyses were two-sided using a level of significance of 0.05. Statistical analyses were performed using IBM SPSS statistics software package version 21 (IBM, USA, 2012). Fractional polynomial analyses were performed in R, version 3.2.0 (R Foundation for Statistical Computing, 2015) using the “mfp” package, version 1.5.1 (2015).

## Results

### Study population

**Fig. 1:**
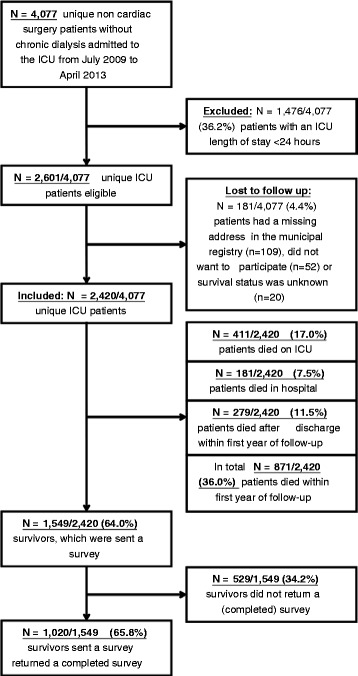
Flowchart. *ICU* intensive care unit

During the inclusion period, 2420 out of 2601 eligible ICU patients were included in this study. One-year survival in the entire population was 1549/2420 patients (64.0 %). Of surviving patients, 1020/1549 (65.8 %) responded to the EQ-5D questionnaire. Hence the outcome status of a total of 1891/2420 (78.0 %) patients one year after ICU admission was known (either alive with known HRQoL (*n* = 1020) or dead (*n* = 871)) (see Fig. 1).

In the total study population, the median age was 59 years (interquartile range (IQR) 47–69) and 1000/2420 patients (41.3 %) were female. The majority of patients, 1418/2420 (58.6 %), were admitted for medical reasons and 1231/2420 patients (50.9 %) had a Charlson Comorbidity Index of one or higher. The median APACHE II score was 19 (IQR 14–25) (see Table 1).

**Table 1:** Patient characteristics in the total population and by RIFLE class

	Total population	RIFLE class				*P* value
		No AKI	Risk	Injury	Failure	
Number	2420	1588 (65.6 %)	456 (18.8 %)	253 (10.5 %)	123 (5.1 %)	
Gender (female)	1000 (41.3 %)	662 (41.7 %)	196 (43 %)	97 (38.3 %)	45 (36.6 %)	.446
Age (years)	59 (47–69)	58 (45–67)	60.5 (50–71)	63 (53–74)	61 (50–72)	<.001
ICU length of stay (days)	3.8 (2–8)	3.4 (2–7)	4.8 (2–9)	4.7 (2–10)	5.3 (2–12)	<.001
Hospital length of stay (days)	17 (8–31)	16.2 (8–29)	18.2 (10–32)	19.9 (8–38)	23.5 (9–40)	.004
Pre-ICU length of stay (days)	0.2 (0–1)	0.2 (0–1)	0.2 (0–1)	0.4 (0–3)	0.4 (0–6)	<.001
Admission type						<.001
Elective surgical	330 (13.6 %)	244 (15.4 %)	47 (10.3 %)	33 (13 %)	6 (4.9 %)	
Urgent surgical	672 (27.8 %)	460 (29 %)	134 (29.4 %)	59 (23.3 %)	19 (15.4 %)	
Medical	1418 (58.6 %)	884 (55.7 %)	275 (60.3 %)	161 (63.6 %)	98 (79.7 %)	
Pre-ICU health state						
Charlson Comorbidity Index	1 (0–2)	0 (0–2)	1 (0–2)	2 (0–4)	2 (0–6)	<0.001
Chronic cardiac insufficiency	217 (9 %)	118 (7.4 %)	50 (11 %)	32 (12.6 %)	17 (13.8 %)	0.003
Chronic respiratory insufficiency or COPD	394 (16.3 %)	229 (14.4 %)	95 (20.8 %)	55 (21.7 %)	15 (12.2 %)	<0.001
Chronic renal insufficiency	96 (4 %)	34 (2.1 %)	12 (2.6 %)	16 (6.3 %)	34 (27.6 %)	<0.001
Mild or severe liver disease	45 (1.9 %)	19 (1.2 %)	7 (1.5 %)	10 (4 %)	9 (7.3 %)	<0.001
Metastatic malignancy	96 (4 %)	53 (3.3 %)	18 (3.9 %)	14 (5.5 %)	11 (8.9 %)	0.016
Haematological malignancy	112 (4.6 %)	54 (3.4 %)	21 (4.6 %)	19 (7.5 %)	18 (14.6 %)	<0.001
HIV positivity, AIDS, or other immunodeficiency	284 (11.7 %)	151 (9.5 %)	56 (12.3 %)	45 (17.8 %)	32 (26 %)	<0.001
Diabetes	341 (14.1 %)	168 (10.6 %)	95 (20.8 %)	54 (21.3 %)	24 (19.5 %)	<0.001
Body mass index	24.7 (22–28)	24.2 (22–27)	25.7 (23–29)	26.1 (23–30)	26 (22–29)	<0.001
Severity of disease markers						
Mechanical ventilation within first 24 hours of ICU stay	2188 (90.4 %)	1426 (89.8 %)	424 (93 %)	230 (90.9 %)	108 (87.8 %)	0.157
Confirmed infection in the first 24 hours of ICU admission	651 (26.9 %)	337 (21.2 %)	136 (29.8 %)	102 (40.3 %)	76 (61.8 %)	<0.001
APACHE II Acute Physiology Score (without creatinine)	17 (13–23)	17 (13–22)	17 (14–23)	17 (14–24)	20 (15–26)	<0.001
APACHE II score (unmodified)	19 (14–25)	18 (14–24)	20 (15–26)	22 (17–28)	29 (23–34)	<0.001
Total maximum SOFA score (sum of highest SOFA component scores)	9 (6–12)	8 (5–11)	10 (7–13)	12 (8–16)	16 (12–18)	<0.001
SOFA score on day of discharge	5 (3–7)	4 (3–6)	5 (3–7)	6 (3–9)	7 (5–13)	<0.001

Values are expressed as number (percentage) for categorical variables and as median (interquartile range) for continuous variables. *RIFLE* risk, injury, failure, loss, end-stage renal failure, *AK*I acute kidney injury, *ICU* intensive care unit; *COPD* chronic obstructive pulmonary disease, *HIV* human immunodeficiency virus, *AIDS* acquired immune deficiency syndrome, *APACHE IV* acute physiology and chronic health evaluation version IV, *SOFA* sequential organ failure assessment

Within the first 24 hours of admission 832/2420 patients fulfilled the RIFLE criteria for risk (456/2420; 18.8 %), injury (253/2420; 10.4 %) or failure (123/2420; 5.1 %). Out of the 123 patients classified as eAKI failure, 62 had renal replacement therapy initiated on the first day of admission. The median time from ICU admission to renal replacement therapy was 7 hours (IQR 4.4–14.5). Except for gender and mechanical ventilation within the first 24 hours of admission, distribution across RIFLE classes differed significantly for all baseline characteristics, with older age, frequent comorbidities and greater disease severity in the RIFLE injury and failure groups (see Table 1).

### Outcomes

Table 2 shows the numbers of patients with one-year poor outcome, mortality, and in one-year survivors, HRQoL. Poor outcome was seen in 1157/2420 subjects (47.8 %) in the total population. In the eAKI subgroups of no eAKI, risk, injury and failure, the incidence of one-year poor outcome was 43.7 % (694/1588), 49.1 % (224/456), 59.7 % (151/253) and 72.4 % (89/123), respectively. Survival rates differed significantly between patients with no AKI and those with increasing severity of eAKI.

**Table 2:** Crude outcomes

	Total population	RIFLE class				*P* value
		No eAKI	Risk	Injury	Failure	
Number at study inclusion	2420	1588	456	253	123	
Composite outcome						
Poor outcome	1157/2420 (47.8 %)	694/1588 (43.7 %)	224/456 (49.1 %)	151/253 (59.7 %)	89/123 (72.4 %)	<0.001
Mortality^a^						
ICU mortality	411 (17 %)	215 (13.5 %)	90 (19.7 %)	65 (25.7 %)	41 (33.3 %)	<0.001
Hospital mortality	592 (24.5 %)	334 (21 %)	117 (25.7 %)	86 (34 %)	55 (44.7 %)	<0.001
One-year mortality	871 (36 %)	503 (31.7 %)	172 (37.7 %)	122 (48.2 %)	74 (60.2 %)	<0.001
Health-related quality of life in one-year survivors						
EQ-5D response	1020/1549 (65.8 %)	735/1085 (67.7 %)	181/284 (63.7 %)	76/131 (58 %)	28/49 (57.1 %)	0.057
EQ-5D index score	0.806 (0.59-0.94)	0.81 (0.64-1.00)	0.778 (0.57-0.89)	0.772 (0.47-0.87)	0.666 (0.37-0.85)	0.076
Low HRQoL^b^	286/1549 (18.5 %)	191/1085 (17.6 %)	52/284 (18.3 %)	29/131 (22.1 %)	15/49 (30.6 %)	0.927

Values are expressed as number (percentage) for categorical variables and as median (interquartile range) for continuous variables. Results were pooled from 35 imputation datasets, using Rubin’s rule to pool statistical test results. ^a^Numbers for ICU, hospital and one-year mortality are cumulative; ^b^Low health related quality of life (HRQoL) was defined as a EuroQoL 5D-3L™ questionnaire (EQ-5D) index below 0.4. RIFLE risk, injury, failure, loss, end-stage renal failure, *eAKI* early acute kidney injury, *ICU* intensive care unit

When compared to the patients who did not experience eAKI, the unadjusted relative risk (RR) for poor outcome was 1.12 (95 % CI 0.99, 1.26; *p* = 0.05) for RIFLE class, risk, 1.36 (95 % CI 1.20, 1.55; *p* < 0.001) for injury and 1.64 (95 % CI 1.34, 2.02; *p* <0.001) for failure. After adjustment for the aforementioned additional set of predictors, the association with poor outcome of RIFLE classes, injury (RR 1.14; 95 % CI 1.01, 1.29; *p* = 0.03) and failure (RR 1.25; 95 % CI 1.01, 1.55; *p* = 0.04) remained statistically significant (see Table 3). Additional file 3 contains separate analyses of the association between eAKI and poor outcome in previously defined ICU subgroups [16]. The association of eAKI with outcome did not differ across subgroups based on admission diagnoses or number of comorbidities. No clear association was seen between eAKI and poor outcome only in patients admitted with traumatic brain injury. eAKI was most frequent in ICU patients with sepsis: 21.6 % of the group of 449 patients (*n* = 97) experienced eAKI risk, 14.3 % (*n* = 64) experienced eAKI injury and 12.0 % experienced eAKI failure (*n* = 54).

**Table 3:**
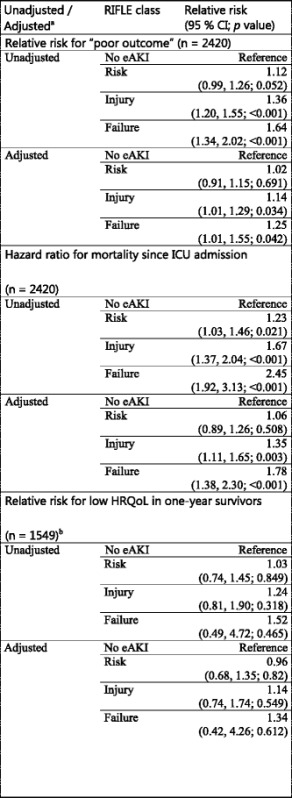
Association eAKI and long-term outcomes

Results were pooled from 35 imputation datasets, using Rubin’s rule. ^a^Adjusted for age, gender, Charlson Comorbidity Index, pre-ICU admission hospital length of stay, admission type, acute physiology score (without creatinine), mechanical ventilation in the first 24 hours of admission and confirmed infection in the first 24 hours of admission. ^b^Low HRQoL was defined as a EuroQoL 5D-3L™ questionnaire (EQ-5D) index below 0.4; age was transformed into ((age-16)/100)^2, APS was transformed into ((APS-1)/10)^-1 + ((APS-1)/10). *APS* Acute Physiology Score, *RIFLE* risk, injury, failure, loss, end-stage renal failure, *eAKI* early acute kidney injury; *ICU* intensive care unit, *HRQoL* health-related quality of life

In the Cox regression analysis, crude estimates of the hazard ratios for mortality in the eAKI RIFLE classes were 1.23 (95 % CI 1.03, 1.46; *p* = 0.02), 1.67 (95 % CI 1.37, 2.04; *p* < 0.001) and 2.45 (95 % CI 1.92, 3.13; *p* < 0.001) for risk, injury and failure. After adjustment for the additional predictors, the hazard ratios for RIFLE classes, injury and failure, remained statistically significant: 1.35 (95 % CI 1.10, 1.65; *p* = 0.004) and 1.77 (95 % CI 1.37, 2.28; *p* < 0.001) (see Table 3). In one-year survivors none of the RIFLE classes were independently associated with low HRQoL (see Table 3).

## Discussion

This cohort study showed that occurrence of AKI early during the ICU stay was associated with an increased probability of being dead or having low HRQoL one year after ICU admission. When compared to patients without eAKI, patients with increasing eAKI severity were associated with increasing risks of poor outcome one year after the ICU stay. Patients with a RIFLE class, failure, on the first day of admission even had a 25 % significantly increased risk of poor outcome, independent of other measured predictors.

To illustrate the effect of eAKI in the setting of the high overall outcome incidence, we used our full statistical model to calculate the absolute predicted probability of poor outcome for two typical ICU patients. Patient A is a low-risk 40-year-old male patient, without comorbidities, admitted to the ICU after elective surgery and a day of prior hospital stay, without an infection or mechanical ventilation within 24 hours of ICU admission and an Acute Physiology Score (APS) of 10. Patient B is a high-risk 60-year-old female patient, with a Charlson Comorbidity Index of 3, admitted to the ICU for medical reasons after a week of prior hospital stay, with a confirmed infection and mechanical ventilation within 24 hours of ICU admission and an APS of 20. If these patients developed severe eAKI (RIFLE failure) Patient A’s risk of poor outcome would increase from 21 to 26 %, while Patient B’s risk would rise from 58 to 72 % (see Table 4 for the full statistical model).

**Table 4:** Poisson regression model for poor outcome

Model		Beta	Relative risk	RR 95 % CI	*P* value
Intercept		−2.401			<0.001
eAKI	No eAKI	Reference			
	Risk	0.023	1.02	0.91, 1.15	0.691
	Injury	0.133	1.14	1.01, 1.29	0.034
	Failure	0.223	1.25	1.01, 1.55	0.042
Sex	Male	Reference			
	Female	0.096	1.10	1.01, 1.2	0.030
Admission type	Elective surgical	Reference			
	Urgent surgical	0.185	1.20	0.99, 1.45	0.057
	Medical	0.270	1.31	1.09, 1.57	0.004
Mechanical ventilation within 24 hours of ICU admission	No	Reference			
	Yes	0.086	1.09	0.93, 1.28	0.300
Confirmed infection within 24 hours of ICU admission	No	Reference			
	Yes	0.116	1.12	1.02, 1.24	0.017
Age (transformed)		1.615	5.03	3.63, 6.96	<0.001
Charlson Comorbidity Index		0.050	1.05	1.03, 1.07	<0.001
Pre-ICU hospital length of stay		0.005	1.01	1.00, 1.01	0.050
Acute Physiology Score (transformed)		0.324	1.38	1.26, 1.52	<0.001

Results were pooled from 35 imputation datasets, using Rubin’s rule. Age was transformed into ((age-16)/100)^2, Acute Physiology Score (APS) was transformed into ((APS-1)/10)^-1 + ((APS-1)/10). *RR 95 % CI* 95 % confidence interval of the relative risk, *eAKI* early acute kidney injury, *ICU* intensive care unit

Patients, family members and clinicians desire more prognostic information about an ICU patient’s survival in conjunction with the expected HRQoL than is currently available [2, 33, 34]. Furthermore, long-term quality of life is conditional on long-term survival. When patients base decisions made during the ICU stay on predicted HRQoL, they need information which also takes into account the condition of long-term survival. We decided to tackle this form of conditionality by creating a composite outcome that is clinically relevant at the time of major ICU treatment decisions. To our knowledge, this is the first study to specifically address this clinically relevant composite endpoint of poor outcome.

So, in respect to the results of previous studies, only the result on the separate constituents of this composite outcome can be compared. The association between (e)AKI and mortality described here is supported by current literature. A recent systematic review described studies of survival for 6 months after ICU discharge. The included studies all reported a large and significant decrease in survival probability in the AKI failure group when compared to all other AKI or no AKI groups [35]. Three studies have reported on the association between (e)AKI and HRQoL in long-term ICU survivors and support the findings presented here. When comparing those survivors who had suffered from (e)AKI and survivors without (e)AKI, there was no significant association with any HRQoL classification [12, 36, 37]. Based on another recent systematic review, the presented study population is by far the largest one to date [38]. Additionally, none of the prior AKI and HRQoL studies took into account the conditionality of HRQoL on survival [12, 36–38]. Finally, with respect to the contribution of survival and HRQoL to the composite endpoint, the increased risk of eAKI for poor outcome seemed to be mainly caused by an increased risk of death within one year after ICU admission.

Different from these previous studies, HRQoL was analysed dichotomously in this study. Aside from this being necessary in order to determine whether a patient suffered from a poor composite outcome, a qualitative interpretation of HRQoL (“low” versus “high” or “severely impaired HRQoL” vs. “not or mildly impaired HRQoL”) was constructed. Choosing a threshold was, and still is, not straightforward. The EQ-5D index itself contained minimal qualitative interpretation: its guidelines merely indicated that a score of 1 corresponds to “full health” and scores below zero to equal states of living valued worse than death [22]. We therefore decided to set a threshold value based on the average EQ-5D index value measured in patients with severe physical, cognitive and/or psychiatric disabilities [23–25]. Still, after classifying patients as such, patients with a low HRQoL might not have considered themselves to be (severely) disabled. However, based on the EQ-5D index formula it can be shown that patients with an EQ-5D index below 0.4 all experienced extreme problems on at least one of the EQ-5D dimensions [22]. Altogether we assumed this threshold therefore corresponded to a clinically relevant major disability or impairment of HRQoL one year after ICU admission.

A strong feature of this study is that we measured and defined RIFLE classification in high detail using an algorithm for routinely collected data. In this study, as originally proposed by Bellomo et al., the RIFLE classification was based on both serum creatinine changes and urine output per hour [5]. As a result, this study distinguished itself from those studies using only serum creatinine changes and/or 24 hour urine output when classifying AKI [35, 39].

Another strength of this study is the way attrition was handled. In cohort studies with lengthy follow up non-response occurs frequently, but seldom completely at random. Consequently, not properly dealing with non-response may lead to bias in any direction by selective loss to follow up [27–30]. In order to minimize the risk of this bias, multiple imputation techniques were used. Additionally, the internal structure of the EQ-5D index was maintained by using these techniques to replace the missing EQ-5D dimensions in survivors who did not respond to the EQ-5D questionnaire, instead of the EQ-5D index value.

However, potential limitations also have to be acknowledged. One limitation of this study is potential unmeasured relevant predictors of poor outcome, and effect modification. In particular, frailty before ICU admission [40] and cardiac or respiratory complications during early ICU admission [41] have recently been suggested as being closely related to, and possibly reducing or altering, the association between AKI and long-term outcomes. As we did not collect data on these variables, it was not possible to account for these factors in our analyses. We did study the predictive values of eAKI in different subgroups (see Additional file 3). These analyses suggested no effect modification or only slight effect modification. Future prognostic studies could study this phenomenon in more detail by accounting for effect modification and frailty in their models.

Another limitation, is that these results apply to the first day of admission only. This might have resulted in an attenuated estimate. The estimate of the effect of eAKI could have been decreased due to patients without early AKI then experiencing AKI later during admission. Data to verify or reject these shortcomings were not available at this time, and this was not the goal of this study. Future research will be aimed at predictors of outcome during the later days of ICU admission.

In clinical practice, some patients and doctors will base their decision for treatment continuation on survival predictions alone, while others decide to incorporate the expected quality of life as the main argument for their treatment wishes. In the process of shared decision-making and accurately informing patients and families, clinicians will then want to provide relevant information [1, 2], without relying on a single predictor for a single outcome. So, given its strong independent association with survival and the composite, poor outcome, which incorporates HRQoL, the severity of eAKI should be considered as a candidate predictor in the future development of multivariable and personalized decision support models, to be used during ICU admission.

## Conclusions

The severity of AKI early during ICU admission was independently associated with increasing risk of one-year poor outcome. In particular, patients with severe eAKI (RIFLE class failure) had a substantially increased risk of poor outcome one year after ICU admission. Together with other early available prognostic factors, information on early acute kidney injury could improve risk-stratification and hence informed decision-making on the continuation or withdrawal of treatment in ICU patients.

## Abbreviations

95 % CI, 95 % confidence interval; AKI, acute kidney injury; APACHE, Acute Physiology and Chronic Health Evaluation; APS, Acute Physiology Score; eAKI, early AKI; EQ-5D, EuroQoL 5D-3L^TM^; HRQoL, health-related quality of life; ICU, intensive care unit; IQR, interquartile range; IRB, Institutional Review Board; RIFLE, risk, injury, failure, loss, end-stage renal failure; RR, relative risk; SAPS, Simplified Acute Physiology Score

## Additional files

Additional file 1: Extended method section. Consists of two sections. The first section contains the definitions used in this study for the Charlson Comorbidity Index and EuroQoL 5D-3L™. The second section contains details about our imputation strategy. (DOCX 38 kb) Additional file 2: Automated RIFLE classification. Consists of a condensed guide on how to replicate our AKI algorithm. Additionally, the associations between the separate parameters to classify RIFLE AKI and poor outcome is shown. (DOCX 117 kb) Additional file 3: Subgroup analysis for poor outcome. Consists of the subgroup analyses for the association of eAKI and poor outcome. (DOCX 32 kb)
