# Supplementary material for: The predictive value of early acute kidney injury for long-term survival and quality of life of critically ill patients
Source: Crit Care. 2016 Aug 3;20:242. doi: 10.1186/s13054-016-1416-0 (PMC4973091; doi:10.1186/s13054-016-1416-0)
Supplement: Additional file 1: — Extended method section. Consists of two sections. The first section contains the definitions used in this study for the Charlson Comorbidity Index and EuroQoL 5D-3L™. The second section contains details about our imputation strategy. (DOCX 38 kb) [file 13054_2016_1416_MOESM1_ESM.docx]

Additional file 1: Extended method section

# Index of additional file

## Definitions

[Charlson comorbidity index page 2](#_Charlson_comorbidity_index)

[EuroQoL 5D-3L^TM^ questionnaire page 3](#_EuroQoL_5D-3LTM_questionnaire)

## MULTIPLE IMPUTATION OF MISSING EQ-5D DIMENSIONS IN NON- OR PARTIAL RESPONDERS

[Description of missing data page 4](#_Description_of_missing)

[Details concerning the imputation technique page 4-5](#_Details_concerning_the)

[Table 2 description of missing data page 6](#_table_2_description)

[Table 3 baseline characteristics in responding and non-responding survivors page 7](#_Table_3_baseline)

[Table 4 distribution of EuroQoL 5D-3LTM dimensions before and after imputation page 8](#_Table_4_distribution)

## Definitions

### Charlson comorbidity index

The Charlson Comorbidity Index is a weighted score based on the presence of 17 comorbidities (chronic diseases in a patient’s past medical history). This index was developed by Charlson and colleagues in 1987 [A]. The weights used for this study were based on a recent update of the Charlson comorbidity index score by Quan and colleagues, performed in 2011 [B]. This recent weighting scheme is available in table 1.

## Table 1 Charlson Comorbidity Index, as weighted by Quan et al.

| Comorbidity | Weight |
| --- | --- |
| Myocardial infarction | 0 |
| Congestive heart failure | 2 |
| Peripheral vascular disease | 0 |
| Cerebrovascular disease | 0 |
| Dementia | 2 |
| Chronic pulmonary disease | 1 |
| Rheumatologic disease | 1 |
| Peptic ulcer disease | 0 |
| Mild liver disease | 2 |
| Diabetes without chronic complications | 0 |
| Diabetes with chronic complications | 1 |
| Hemiplegia or paraplegia | 2 |
| Renal disease | 1 |
| Any malignancy, including leukaemia and lymphoma | 2 |
| Moderate or severe liver disease | 4 |
| Metastatic solid tumour | 6 |
| AIDS/HIV | 4 |

Adapted from Quan H, Li B, Couris CM, Fushimi K, Graham P, Hider P, Januel JM, Sundararajan V. Updating and validating the Charlson comorbidity index and score for risk adjustment in hospital discharge abstracts using data from 6 countries. Am J Epidemiol. 2011 Mar 15;173(6):676-82. doi: 10.1093/aje/kwq433.

### EuroQoL 5D-3L^TM^ questionnaire

HRQoL was measured using the EuroQoL 5D-3LTM questionnaire (EQ-5D). This questionnaire consists of five questions each representing a dimension of HRQoL. The dimensions are mobility, self-care, usual activities, pain or discomfort, anxiety or depression. Patients assigned a score of no (1), little (2) or many problems (3) to each of these dimensions. Based on these five dimensions with 3 possible answers each, 35 (243) health states can be discerned. For many different countries the EuroQoL group has studied how the differing populations value certain health states. Out of these studies the EuroQoL group developed so-called national tariffs which put these health states on a scale from “1” (full health) to “0” (dead). Negative values, or health states valued worse than death, were also possible. This scale is the EQ-5D index scale. For this study, the subjects’ answers to the EQ-5D’s 5 dimensions were indexed according to the weighting scheme for the Dutch population [C].

For further reference please visit the EuroQoL organisation’s website at <http://www.euroqol.org/>

## Multiple Imputation of missing EQ-5D dimensions in non- or partial responders

### Description of missing data

For data on RIFLE and the additional predictors, great care was taken to have complete data. This was done by retrieving all required data from electronic patient files, referring back to these files by hand search where missings occurred. Data on additional variables were also retrieved (see ‘Rationale and methods for the multiple imputation technique’ below). Not all of these additional variables could be retrieved. For 120/1,549 (7.7%) patients data on SOFA scores during admission were missing, while APACHE IV score at admission was missing in 98/1,549 (6.3%) patients.

Outcome data on survival was complete for all 2,420 included patients. In this study, 532/1,549 (34.3%) patients did not respond to the EuroQoL 5D-3L^TM^ questionnaire (EQ-5D) questionnaire or returned only a partially completed one. As no EQ-5D index score could be calculated, these subjects had a missing outcome status for the composite outcome. See Appendix table 2 for more details on missingness of data per patient characteristic. Differences in baseline characteristics between responders and non-responders are shown in Appendix table 3.

Responders and non-responders differed significantly for age, mild or severe liver disease and diabetes. As this may lead to biased effect estimates, multiple imputation techniques have been used to impute missing values [D-H]. This was done under the assumption of data being missing at random.

### Details concerning the imputation technique

The following variables were used as predictors within the imputation model: RIFLE classification, age, gender, pre-ICU hospital length of stay, admission type, Charlson comorbidity index and severity of disease markers such as need for mechanical ventilation, confirmed infection and acute physiology score (without creatinine) in the first 24 hours of admission, body mass index, specific chronic comorbidities (congestive heart failure; HIV, AIDS or immunodeficiency; chronic pulmonary disease; chronic renal disease; chronic liver disease; and diabetes), APACHE-IV score, SOFA score sum of maximum component scores during ICU admission, SOFA score at day of discharge, ICU and hospital length of stay, and the survival outcome were also entered into the imputation model.

Rounding and boundaries were used to assure replaced missing values of continuous variables yielded clinically possible values. As the maximum percentage of missing data was 34% (non-response on the 1year QoL questionnaire), 35 imputation datasets were created.

As patients who died within one year after ICU admission were different from survivors with regards to their disease characteristics and the association with HRQoL, only the data of the responders to the questionnaire was used to impute missing data in non-responding survivors.

Within the multiple imputation procedure, EQ-5D dimensions were set as ordinal variables with three possible values (none/mild/extreme problems, respectively coded as ‘1’/’2’/’3’). The imputation model used multivariable logistic multinomial regression to replace these missing data.

After the imputation datasets were created, the EQ-5D index score was calculated for each patient in each dataset using the Dutch weighting scheme. Finally, EQ-5D index score was dichotomized and used in accordance with survival to create the composite outcome measure. Results of analyses in the different imputation datasets were pooled using Rubin’s rule to arrive at correct effect estimates and standard errors [D,H]. Data handling, analysis, multiple imputation and pooling of results were performed using IBM SPSS statistics software package version 21 (IBM, United States of America, 2012) and R, version 3.2.0 (R Foundation for Statistical Computing, 2015) using the ‘miceadds’ package, version 1.4.0 (2015). For all analyses the pooled results were considered the primary results of this study.[H]

Table 4 shows the distribution of EQ-5D dimensions before imputation in complete cases and after imputation. The results of the 35 datasets are pooled and rounded to the nearest integer. Compared to complete case analysis, after imputation a larger proportion of patients suffered from moderate or extreme disabilities on all five EQ-5D dimensions.

## References

[A] Charlson ME, Pompei P, Ales KL, MacKenzie CR. A new method of classifying prognostic comorbidity in longitudinal studies: development and validation. J Chronic Dis. 1987;40(5):373-83.

[B] Quan H, Li B, Couris CM, Fushimi K, Graham P, Hider P, Januel JM, Sundararajan V. Updating and validating the Charlson comorbidity index and score for risk adjustment in hospital discharge abstracts using data from 6 countries. Am J Epidemiol. 2011 Mar 15;173(6):676-82. doi: 10.1093/aje/kwq433.

[C] Szende A, Oppe M, de Charro F. Chapter 2 Comparative review of Time-Trade-Off value sets. In: Szende A, Oppe M, Devlin N, Eds.: EQ-5D Value Sets: Inventory, Comparative Review and User Guide. Springer, Dordrecht, 2007. p. 21-27

[D] Rubin DB. Multiple imputation for non response in surveys. Wiley, New York 1987

[E] Donders ART, van der Heijden GJMG, Stijnen T, Moons KGM. Review: a gentle introduction to imputation of missing values. Journal of clinical epidemiology 2006;59(10):1087–91

[F] Li P, Stuart EA, Allison DB. Multiple Imputation: A Flexible Tool for Handling Missing Data. JAMA 2015 Nov 10;314(18):1966-7. doi: 10.1001/jama.2015.15281

[G] Groenwold RHH, Donders ART, Roes KCB, Harrell FE, Moons KGM. Dealing with missing outcome data in randomized trials and observational studies. American journal of epidemiology 2012;175(3):210–7. doi: 10.1093/aje/kwr302

[H] Van Buuren S, Groothuis-oudshoorn K. mice: Multivariate Imputation by Chained Equations in R. Journal Of Statistical Software 2011;45(3).

## Table 2 description of missing data

|  |  | Missing, n (%) |
| --- | --- | --- |
| Determinants included in analysis | Gender | 0/1,549 (0%) |
|  | Age | 0/1,549 (0%) |
|  | Pre-ICU hospital length of stay | 0/1,549 (0%) |
|  | Admission type | 0/1,549 (0%) |
|  | CCI | 0/1,549 (0%) |
|  | Acute Physiology Score (without creatinine) | 0/1,549 (0%) |
|  | Mechanical ventilation within first 24 hours of ICU stay | 0/1,549 (0%) |
|  | Confirmed infection in the first 24 hours of ICU admission | 0/1,549 (0%) |
|  | RIFLE classification | 0/1,549 (0%) |
|  |  |  |
| Additional determinants for multiple imputation | Cardiac insufficiency or congestive heart failure | 0/1,549 (0%) |
|  | Chronic respiratory insufficiency or COPD | 0/1,549 (0%) |
|  | Chronic renal insufficiency | 0/1,549 (0%) |
|  | Mild or severe liver disease | 0/1,549 (0%) |
|  | Metastatic malignancy | 0/1,549 (0%) |
|  | Haematological malignancy | 0/1,549 (0%) |
|  | HIV positivity, AIDS or other immunodeficiency | 0/1,549 (0%) |
|  | Diabetes | 0/1,549 (0%) |
|  | Body Mass Index | 1/1,549 (0.1%) |
|  | Total maximum SOFA score | 120/1,549 (7.7%) |
|  | SOFA score on day of discharge | 120/1,549 (7.7%) |
|  | ICU length of stay | 0/1,549 (0%) |
|  | Hospital length of stay | 0/1,549 (0%) |
|  | APACHE IV score | 97/1,549 (6.3%) |
|  |  |  |
| Outcomes | Death at 1 year follow-up | 0/1,549 (0%) |
|  | EQ-5D index value | 529/1,549 (34.2%) |
|  | Poor HRQoL | 529/1,549 (34.2%) |
|  | Composite outcome | 529/1,549 (34.2%) |
|  |  |  |
| EQ-5D dimensions | Mobility | 508/1,549 (32.8%) |
|  | Self-care (e.g. like washing or dressing yourself) | 509/1,549 (32.9%) |
|  | Usual Activities (e.g. occupation, study, housekeeping, family- or leisure activities) | 508/1,549 (32.8%) |
|  | Pain/Discomfort | 511/1,549 (33%) |
|  | Anxiety/Depression | 505/1,549 (32.6%) |

ICU, intensive care unit; CCI, Charlson comorbidity index; COPD, chronic obstructive pulmonary disease; HIV, human immunodeficiency virus; AIDS, acquired immune deficiency syndrome; SOFA, sequential organ failure assessment; APACHE IV, acute physiology and chronic health evaluation version IV; EQ-5D, EuroQoL 5D-3LTM questionnaire.

## Table 3 baseline characteristics in responding and non-responding survivors

|  | **Responders** | **Non-responders** | **p-value** | **Deceased** |
| --- | --- | --- | --- | --- |
| **N** | 1,020 | 529 |  | 871 |
|  |  |  |  |  |
| Gender (female) | 417 (40.9%) | 227 (42.9%) | .447 | 356 (40.9%) |
| Age (years) * | 58 (46 - 66) | 52 (38 - 64) | .000 | 64 (53 - 74) |
| ICU length of stay (days) | 3.6 (2 - 7) | 3.7 (2 - 7) | .904 | 4.4 (2 - 9) |
| Hospital length of stay (days) | 19.6 (12 - 32) | 18.2 (10 - 34) | .114 | 12.1 (5 - 27) |
| Pre-ICU length of stay (days) | 0.2 (0 - 1) | 0.2 (0 - 1) | .682 | 0.2 (0 - 3) |
|  |  |  |  |  |
| **Admission type** |  |  | .271 |  |
| elective surgical | 167 (16.4%) | 84 (15.9%) |  | 79 (9.1%) |
| urgent surgical | 312 (30.6%) | 143 (27%) |  | 217 (24.9%) |
| medical | 541 (53%) | 302 (57.1%) |  | 575 (66%) |
|  |  |  |  |  |
| **Comorbidities** |  |  |  |  |
| CCI | 0 (0 - 2) | 0 (0 - 2) | .278 | 2 (0 - 3) |
| Cardiac insufficiency or congestive heart failure | 86 (8.4%) | 42 (7.9%) | .771 | 89 (10.2%) |
| Chronic respiratory insufficiency or COPD | 161 (15.8%) | 94 (17.8%) | .348 | 139 (16%) |
| Chronic renal insufficiency | 28 (2.7%) | 13 (2.5%) | .744 | 55 (6.3%) |
| Mild or severe liver disease | 11 (1.1%) | 12 (2.3%) | .077 | 22 (2.5%) |
| Metastatic malignancy | 34 (3.3%) | 10 (1.9%) | .110 | 52 (6%) |
| Haematological malignancy | 24 (2.4%) | 7 (1.3%) | .186 | 81 (9.3%) |
| HIV positivity. AIDS or other immunodeficiency | 95 (9.3%) | 49 (9.3%) | .999 | 140 (16.1%) |
| Diabetes * | 106 (10.4%) | 89 (16.8%) | .000 | 146 (16.8%) |
| Body Mass Index | 24.8 (22 - 28) | 24.8 (22 - 28) | .924 | 24.6 (22 - 28) |
|  |  |  |  |  |
| **RIFLE** |  |  | .057 |  |
| No AKI | 735 (72.1%) | 350 (66.2%) |  | 503 (57.7%) |
| Risk | 181 (17.7%) | 103 (19.5%) |  | 172 (19.7%) |
| Injury | 76 (7.5%) | 55 (10.4%) |  | 122 (14%) |
| Failure | 28 (2.7%) | 21 (4%) |  | 74 (8.5%) |
|  |  |  |  |  |
| **Severity of disease markers** |  |  |  |  |
| Mechanical ventilation within first 24 hours of ICU stay | 905 (88.7%) | 483 (91.3%) | .135 | 800 (91.8%) |
| Confirmed infection in the first 24 hours of ICU admission | 232 (22.7%) | 122 (23.1%) | .899 | 297 (34.1%) |
| Acute Physiology Score (without creatinine) | 16 (12 - 20) | 16 (12 - 21) | .830 | 21 (15 - 27) |
| APACHE IV score | 55 (40 - 74) | 53 (37 - 73) | .135 | 83 (63 - 109) |
| Total maximum SOFA score (sum of highest SOFA component scores) | 8 (5 - 11) | 8 (5 - 11) | .618 | 11 (8 - 14) |
| SOFA score on day of discharge | 4 (2 - 5) | 4 (3 - 5) | .777 | 7 (4 - 11) |

Values are expressed as number (percentage) for categorical variables and as median (interquartile range) for continuous variables. *, significant difference of distribution (p<.05) tested between responders and non-responders; AKI, acute kidney injury; ICU, intensive care unit; CCI, Charlson comorbidity index; COPD, chronic obstructive pulmonary disease; HIV, human immunodeficiency virus; AIDS, acquired immune deficiency syndrome; APACHE IV, acute physiology and chronic health evaluation version IV; SOFA, sequential organ failure assessment.

## Table 4 distribution of EuroQoL 5D-3LTM dimensions before and after imputation

|  |  | Before imputation (n=1,020) | After imputation, pooled (n=1,549) |
| --- | --- | --- | --- |
| **Mobility** | I have no problems walking about | 614/1,020 (60.2%) | 907/1,549 (58.6%) |
|  | I have some problems walking about | 367/1,020 (36%) | 577/1,549 (37.2%) |
|  | I am confined to bed | 39/1,020 (3.8%) | 65/1,549 (4.2%) |
|  |  |  |  |
| **Self-care (e.g. like washing or dressing yourself)** | I have no problems with self-care | 773/1,020 (75.8%) | 1151/1,549 (74.3%) |
|  | I have some problems washing or dressing myself | 162/1,020 (15.9%) | 249/1,549 (16.1%) |
|  | I am unable to wash or dress myself | 85/1,020 (8.3%) | 149/1,549 (9.6%) |
|  |  |  |  |
| **Usual Activities (e.g. occupation, study, housekeeping, family- or leisure activities)** | I have no problems with performing my usual activities | 438/1,020 (42.9%) | 643/1,549 (41.5%) |
|  | I have some problems in with performing my usual activities | 442/1,020 (43.3%) | 678/1,549 (43.8%) |
|  | I am unable to perform my usual activities | 140/1,020 (13.7%) | 228/1,549 (14.7%) |
|  |  |  |  |
| **Pain/Discomfort** | I have no pain or discomfort | 514/1,020 (50.4%) | 767/1,549 (49.5%) |
|  | I have moderate pain or discomfort | 455/1,020 (44.6%) | 687/1,549 (44.4%) |
|  | I have extreme pain or discomfort | 51/1,020 (5%) | 95/1,549 (6.1%) |
|  |  |  |  |
| **Anxiety/Depression** | I am not anxious or depressed | 721/1,020 (70.7%) | 1075/1,549 (69.4%) |
|  | I am moderately anxious or depressed | 261/1,020 (25.6%) | 392/1,549 (25.3%) |
|  | I am extremely anxious or depressed | 38/1,020 (3.7%) | 82/1,549 (5.3%) |
