# Supplementary material for: The predictive value of early acute kidney injury for long-term survival and quality of life of critically ill patients
Source: Crit Care. 2016 Aug 3;20:242. doi: 10.1186/s13054-016-1416-0 (PMC4973091; doi:10.1186/s13054-016-1416-0)
Supplement: Additional file 2: — Automated RIFLE classification. Consists of a condensed guide on how to replicate our AKI algorithm. Additionally, the associations between the separate parameters to classify RIFLE AKI and poor outcome is shown. (DOCX 117 kb) [file 13054_2016_1416_MOESM2_ESM.docx]

Additional file 2. Automated RIFLE classification

# Index of additional file

## Data handling

[Serum creatinine page 2](#_Serum_creatinine)

[Start of renal replacement therapy page 2](#_Start_of_renal)

[Urine output page 3-5](#_Urine_output)

[RIFLE scoring page 5](#_RIFLE_scoring)

## Association between separate RIFLE classifications and poor outcome

[Results of analysis page 6-8](#_Association_between_separate)

# Data handling

Data handling for RIFLE classification was split up into three different parts upon which RIFLE was classified: serum creatinine, start of renal replacement therapy and, the most elaborate part, urine output.

### Serum creatinine

Serum creatinine based RIFLE classification is based on changes over time. So, to score AKI based on creatinine (RIFLEcreat) at least two measurements were necessary. As estimation of serum creatinine using MDRD-formula is unreliable in critically ill patients, we used an actually measured serum creatinine level as baseline. The baseline was defined as the lowest serum creatinine levels in the six months prior to ICU admission. These were obtained from our hospital laboratory data systems. If no prior-ICU baseline serum creatinine level was available, the lowest serum creatinine during the first 24 hours of ICU admission was used as baseline creatinine. The highest serum creatinine level measured during the first 24 hours was used to calculate the factor of creatinine change. Factors of change higher than 1.5, 2.0 or 3.0 were respectively scored as RIFLE ‘Risk’, ‘Injury’ or ‘Failure’. Additionally, patients in which the highest serum creatinine level was higher than 350μmol/l and an absolute increase of equal to or more than 44μmol/l occurred (acute-on-chronic kidney failure) were also scored as ‘Failure’.

For patients with no known baseline measurement and only one ICU serum creatinine measurement, no serum creatinine change was calculable and these patients were considered not to have AKI based on creatinine values. In patients with no available creatinine levels, RIFLEcreat could not be scored (i.e. RIFLEcreat was considered missing).

When data were handled this way, a pre-ICU creatinine was available in 1,217/2,420 (50.3%) as baseline. In-ICU serum creatinine levels were available in 2,364/2,420 (97.7%) patients. Including the pre-ICU baseline creatinine level as a measurement, 2,143/2,420 (88.6%) patients had two or more creatinine measurements in the first 24 hours of admission. Finally, RIFLEcreat was classifiable in 2361/2420 (97.6%) patients.

### Start of renal replacement therapy

Renal replacement therapy (RRT) was scored during each nurse’s shift (once every 8 hours, as part of registration of invasive clinical treatments). Furthermore, the dialysis devices used on our unit fed the electronic patient file with information on its use.

For a patient to be scored as ‘on RRT’ on the first day of ICU admission, the dialysis device needed to return a ‘Running’ status with a flow of blood going through the apparatus of more than 0ml/min. For any other device state, or a flow of blood of 0ml/min patients were scored as ‘not on RRT’. If data from a dialysis device was missing and patients were not scored as ‘on RRT’ by the nurses, patients were considered ‘not on RRT’. In the case data from a dialysis device was missing and patients were scored as on dialysis by the nurses, a case study was performed to check for the actual use of dialysis. This was necessary in 7/2,420 (0.3%) patients. Any patient on RRT was scored as RIFLE ‘Failure’ based on RRT use (RIFLERRT), while any patient not on RRT was scored as ‘no AKI’ based on RRT use.

### Urine output

For UO, data was extracted from the ICU electronic patient file. Nurses manually registered UO on an hourly basis for clinical monitoring. RIFLE based on UO (RIFLEurine) was scored according to the following periods of oliguria, adjusted for patient weight at admission: patients with an UO less than 0.5ml/kg/hour for six hours; twelve hours; or less than 0.3ml/kg/hour for 24 hours were respectively scored as RIFLE ‘Risk’, ‘Injury’ and ‘Failure’. Additionally patients with anuria (urine production of 0ml/kg/hour) for twelve hours were also scored as RIFLE ‘Failure’ (see table 1,2 and 3 for examples). The first measurement was disregarded for RIFLE classification to exclude influence from unmeasured factors prior to ICU admission.

## Table 1

| Hour of admission | 0 | 1 | 2 | 3 | 4 | 5 | 6 | 7 | 8 | 9 | 10 | 11 | 12 | 13 | 14 | 15 | 16 | 17 | 18 | 19 | 20 | 21 | 22 | 23 | 24 |
| --- | --- | --- | --- | --- | --- | --- | --- | --- | --- | --- | --- | --- | --- | --- | --- | --- | --- | --- | --- | --- | --- | --- | --- | --- | --- |
| Urine production (ml) | 60 | 55 | 50 | 45 | 40 | 35 | 30 | 30 | 25 | 20 | 30 | 45 | 50 | 50 | 50 | 50 | 70 | 50 | 50 | 50 | 60 | 50 | 50 | 50 | 50 |
| Patient weight = 80kg with RIFLE ‘Risk’ in the fifth up to the tenth hour of admission (urine output below 0.5ml/kg/hour = <40ml/hour, for at least six and less than twelve consecutive hours). The first measurement has been greyed out to show it is not used for RIFLE classification. | | | | | | | | | | | | | | | | | | | | | | | | | |

## Table 2

| Hour of admission | 0 | 1 | 2 | 3 | 4 | 5 | 6 | 7 | 8 | 9 | 10 | 11 | 12 | 13 | 14 | 15 | 16 | 17 | 18 | 19 | 20 | 21 | 22 | 23 | 24 |
| --- | --- | --- | --- | --- | --- | --- | --- | --- | --- | --- | --- | --- | --- | --- | --- | --- | --- | --- | --- | --- | --- | --- | --- | --- | --- |
| Urine production (ml) | 60 | 55 | 50 | 45 | 40 | 45 | 45 | 50 | 40 | 20 | 30 | 50 | 25 | 20 | 15 | 10 | 10 | 10 | 10 | 15 | 20 | 15 | 10 | 10 | 5 |
| Patient weight = 80kg with RIFLE ‘Injury’ in the twelfth up to the twenty-fourth hour of admission (urine output below 0.5ml/kg/hour = <40ml/hour for at least twelve consecutive hours). Hour 9 and 10 were also below the threshold of 40ml/hour, but these were not included in the stretch used for RIFLE classification because of the urine production in hour 11 was above 40ml/hour (not consecutive). | | | | | | | | | | | | | | | | | | | | | | | | | |

## Table 3

| Hour of admission | 0 | 1 | 2 | 3 | 4 | 5 | 6 | 7 | 8 | 9 | 10 | 11 | 12 | 13 | 14 | 15 | 16 | 17 | 18 | 19 | 20 | 21 | 22 | 23 | 24 |
| --- | --- | --- | --- | --- | --- | --- | --- | --- | --- | --- | --- | --- | --- | --- | --- | --- | --- | --- | --- | --- | --- | --- | --- | --- | --- |
| Urine production (ml) | 25 | 20 | 20 | 20 | 10 | 15 | 20 | 15 | 20 | 15 | 20 | 5 | 0 | 0 | 0 | 0 | 0 | 0 | 0 | 0 | 0 | 0 | 0 | 0 | 0 |
| Patient weight = 80kg with RIFLE ‘Failure’ (urine output below 0.3ml/kg/hour = <24ml/hour during 24 consecutive hours). Again, the first measurement at admission was deleted because of unknown collection time for this measurement, possibly occurring before ICU admission. Because the following and actual first 24 hours of admission measurements were known, excluding this measurement did not result in a RIFLE misclassification. Secondly, this patient also experienced at least twelve hours of anuria, also resulting in RIFLE ‘Failure’ classification. | | | | | | | | | | | | | | | | | | | | | | | | | |

Regrettably, data on UO was not complete in all cases. Only in 462/2,420 (19.1%) cases all 24 measurements of the first day of admission were recorded. However, including those with complete data, only six measurements or less were missing in 2,083/2,420 (86.1%) subjects. Moreover, only 147/2,420 (6.1%) patients had stretches of consecutive missing UO measurements of more than six hours, while only 41/2,420 (1.7%) patients had stretches of consecutive missing UO measurements of more than twelve hours.

We believe these numbers of missing represent common clinical practice, where, despite lacking some measurements, clinicians are still able to make judgments about the presence of AKI based on urine output. A clinician trying to estimate kidney functioning based on UO could divide the amount of UO registered by the amount of time in between the measurements to estimate hourly UO and kidney functioning. Estimating the hourly UO for all 24 hours of the first day in case of missing values can be performed by calculating the time, in hours, of the missing stretch plus one for the first known measurement, and dividing the amount of UO in the first known measurement by this calculated time-gap (see tables 4A,B for an example). For further reference, we have named this process of distributing UO over a missing period of time ‘backlogging’.

## Table 4a

| Hour of admission | 1 | 2 | 3 | 4 | 5 | 6 | 7 | 8 | 9 | 10 | 11 | 12 | 13 | 14 | 15 | 16 | 17 | 18 | 19 | 20 | 21 | 22 | 23 | 24 | 25 |
| --- | --- | --- | --- | --- | --- | --- | --- | --- | --- | --- | --- | --- | --- | --- | --- | --- | --- | --- | --- | --- | --- | --- | --- | --- | --- |
| Urine production (ml) | 55 | 50 | 45 | M | M | 105 | 30 | 25 | 20 | 30 | M | 96 | 50 | 50 | 50 | 50 | 55 | 50 | 50 | 50 | M | M | M | M | 250 |
| Patient weight = 80kg with missing urine output values (M), before backlogging. Without backlogging, this patient would not be scored as suffering from AKI as no consecutive stretch of six or more hours was below 0.5ml/kg/hour = <40ml/hour. The first measurement has been left out of this table) | | | | | | | | | | | | | | | | | | | | | | | | | |

## Table 4b

| Hour of admission | 1 | 2 | 3 | 4 | 5 | 6 | 7 | 8 | 9 | 10 | 11 | 12 | 13 | 14 | 15 | 16 | 17 | 18 | 19 | 20 | 21 | 22 | 23 | 24 | 25 |
| --- | --- | --- | --- | --- | --- | --- | --- | --- | --- | --- | --- | --- | --- | --- | --- | --- | --- | --- | --- | --- | --- | --- | --- | --- | --- |
| Urine production (ml) | 55 | 50 | 45 | 35 | 35 | 35  105/3 | 30 | 25 | 20 | 30 | 48 | 48  96/2 | 50 | 50 | 50 | 50 | 55 | 50 | 50 | 50 | 50 | 50 | 50 | 50 | 50  250/5 |
| Patient weight = 80kg, after backlogging. This patient is now scored RIFLE ‘Risk’ from the fourth up to the tenth hour of admission (urine output below 0.5ml/kg/hour = <40ml/hour, for at least six and less than twelve hours), while the other values backlogged this way were not indicative of AKI. Also, the twenty-fifth hour of admission was also used to backlog the last hours of the first day of admission. | | | | | | | | | | | | | | | | | | | | | | | | | |

It is possible to create a dataset, where no missing UO measurements occur, while using backlogging. However, we were afraid this might lead to over- and misclassification of RIFLEurine. See for example tables 5a and 5b where backlogging led to an AKI classification.

## Table 5a

| Hour of admission | 1 | 2 | 3 | 4 | 5 | 6 | 7 | 8 | 9 | 10 | 11 | 12 | 13 | 14 | 15 | 16 | 17 | 18 | 19 | 20 | 21 | 22 | 23 | 24 | 25 |
| --- | --- | --- | --- | --- | --- | --- | --- | --- | --- | --- | --- | --- | --- | --- | --- | --- | --- | --- | --- | --- | --- | --- | --- | --- | --- |
| Urine production (ml) | M | M | M | M | M | M | M | M | M | M | M | M | M | M | 300 | 60 | 70 | 55 | 60 | 70 | 55 | 60 | 55 | 50 | 60 |
| Patient weight = 80kg with missing urine output values (M), before backlogging, where based on the known hours no RIFLE AKI can be scored. | | | | | | | | | | | | | | | | | | | | | | | | | |

## Table 5b

| Hour of admission | 1 | 2 | 3 | 4 | 5 | 6 | 7 | 8 | 9 | 10 | 11 | 12 | 13 | 14 | 15 | 16 | 17 | 18 | 19 | 20 | 21 | 22 | 23 | 24 | 25 |
| --- | --- | --- | --- | --- | --- | --- | --- | --- | --- | --- | --- | --- | --- | --- | --- | --- | --- | --- | --- | --- | --- | --- | --- | --- | --- |
| Urine production (ml) | 20 | 20 | 20 | 20 | 20 | 20 | 20 | 20 | 20 | 20 | 20 | 20 | 20 | 20 | 20  300/15 | 60 | 70 | 55 | 60 | 70 | 55 | 60 | 55 | 50 | 60 |
| Patient weight = 80kg, after backlogging. Note that this patient is now scored RIFLE ‘Injury’ from the first up and including to the fifteenth hour of admission (urine output below 0.5ml/kg/hour = <40ml/hour, for more than twelve hours). | | | | | | | | | | | | | | | | | | | | | | | | | |

Aside from a lack of monitoring, the possible reasons for similar consecutive stretches of missing values could range from being simple to extremely difficult to explain based on data available in electronic patient files.

Restricting the backlogging process was our solution to this problem. We incorporated this by backlogging only up to a maximum of six hours. This maximum ‘latency’ of six hours was chosen for multiple reasons. The most important one being that using the six hours maximum latency period ensures one UO measurement is not stretched beyond the confines of one period of time needed to score AKI when using RIFLE.

Revisiting the case from table 5, using restricted backlogging now shows no AKI in the known or backlogged UP measurements:

## Table 5c

| Hour of admission | 1 | 2 | 3 | 4 | 5 | 6 | 7 | 8 | 9 | 10 | 11 | 12 | 13 | 14 | 15 | 16 | 17 | 18 | 19 | 20 | 21 | 22 | 23 | 24 | 25 |
| --- | --- | --- | --- | --- | --- | --- | --- | --- | --- | --- | --- | --- | --- | --- | --- | --- | --- | --- | --- | --- | --- | --- | --- | --- | --- |
| Urine production (ml) | M | M | M | M | M | M | M | M | M | 50 | 50 | 50 | 50 | 50 | 50  300/6 | 60 | 70 | 55 | 60 | 70 | 55 | 60 | 55 | 50 | 60 |
| Patient weight = 80kg, after backlogging. This patient is now scored no AKI (not one hour where urine production was below 0.5ml/kg/hour = <40ml/hour). There are still missing measurements remaining, but based on known and backlogged UP measurements, this patient did not suffer from AKI. | | | | | | | | | | | | | | | | | | | | | | | | | |

We formulated that for patients with twelve or more missing UP measurements, after backlogging, RIFLEurine could not be scored (i.e. RIFLEurine was considered missing). RIFLE classification would then depend on creatinine changes or the use of renal replacement therapy.

When data on UP were handled this way, RIFLEurine was available in 2,386/2,420 (98.6%) patients in our study cohort.

### RIFLE scoring

Combining all three aspects (RIFLE_creat_, RIFLE_RRT_ and RIFLE_urine_) into a final RIFLE classification was done by taking the highest/most severe RIFLE class from either one of these parts as the final RIFLE classification.

Handling data this way resulted in RIFLE classification in 2,412/2,420 (99.7%) patients. For the remaining eight patients, charts were checked to reveal a no AKI state on the first day of ICU admission for all eight of them.

# Association between separate RIFLE classifications and poor outcome

To assess the validity of our RIFLE scoring system, the association between the RIFLE component classifications and the composite outcome of survival and were examined and provided in this additional file. The same methods were used as used for the analyses described in the main text.

Figure 1 shows the uni- and multivariable association between the separate RIFLE classifications and a poor outcome. The univariable analyses showed both RIFLE_creat_ and RIFLE_urine_ are associated with unfavourable outcome with increasing severity. However, in the multivariable analysis for RIFLE_creat_ only the presence of small creatinine increases on the first day of admission were significantly associated with unfavourable outcome. In the multivariable analysis for RIFLE_urine_, increasing severity of eAKI remained associated with higher risks of poor outcome.

**Table 1 relative risk for unfavourable outcome using Poisson regression**

| RIFLE based on serum creatinine changes  (n= 2,361/2,420; 97.6%) | Unadjusted / Adjusted a | RIFLE class | Relative risk  (95%CI; p-value) |
| --- | --- | --- | --- |
|  | Unadjusted | No eAKI | Reference |
|  |  | Risk | 1.37  (1.19-1.58; <.001) |
|  |  | Injury | 1.41  (1.18-1.68; <.001) |
|  |  | Failure | 1.56  (1.24-1.96; <.001) |
|  | Adjusted | No eAKI | Reference |
|  |  | Risk | 1.17  (1.01-1.34; .031) |
|  |  | Injury | 1.08  (0.90-1.31; .397) |
|  |  | Failure | 1.17  (0.91-1.50; .228) |
| RIFLE based on hourly urine output  (n=2,386/2,420; 98.6%) | Unadjusted / Adjusted ^a^ | RIFLE class | Relative risk  (95%CI; p-value) |
|  | Unadjusted | No eAKI | Reference |
|  |  | Risk | 1.08  (0.96-1.22; .223) |
|  |  | Injury | 1.40  (1.24-1.58; <.001) |
|  |  | Failure | 1.77  (1.47-2.13; <.001) |
|  | Adjusted | No eAKI | Reference |
|  |  | Risk | 1.00  (0.89-1.12; .959) |
|  |  | Injury | 1.13  (1.00-1.27; .051) |
|  |  | Failure | 1.29  (1.06-1.56; .011) |
|  |  |  |  |
| Rifle based on initiation of RRT  (n=2420/2420; 100%) | Unadjusted / Adjusted a | RIFLE class | Relative risk  (95%CI; p-value) |
|  | Unadjusted | No eAKI | Reference |
|  |  | RRT initiated within 24 hours (Failure) | 1.43 (1.09-1.88; .009 |
|  | Adjusted | No eAKI | Reference |
|  |  | RRT initiated within 24 hours (Failure) | 1.17 (0.89-1.53; .262) |

Results were pooled from 35 imputation datasets, using Rubin’s rule. a, adjusted for age, gender, Charlson comorbidity index, pre-intensive care unit admission hospital length of stay, admission type, acute physiology score (without creatinine), mechanical ventilation in the first 24 hours of admission and confirmed infection in the first 24 hours of admission. Age was transformed into ((age-16)/100)^2, APS was transformed into ((APS-1)/10)^-1+((APS-1)/10); 95%CI, 95% confidence interval; eAKI, early acute kidney injury;
