# Supplementary material for: The predictive value of early acute kidney injury for long-term survival and quality of life of critically ill patients
Source: Crit Care. 2016 Aug 3;20:242. doi: 10.1186/s13054-016-1416-0 (PMC4973091; doi:10.1186/s13054-016-1416-0)
Supplement: Additional file 3: — Subgroup analysis for poor outcome. Consists of the subgroup analyses for the association of eAKI and poor outcome. (DOCX 32 kb) [file 13054_2016_1416_MOESM3_ESM.docx]

Additional file 3. Subgroup analysis for poor outcome

# Index of additional file

## Subgroup Analysis

[Table 1 admission diagnosis subgroup analysis of eAKI for poor outcome page 2](#_Table_1_Admission)

[Box 1. Subgroup definitions page 3-4](#_Box_1._Subgroup)

[Table 2 amount of comorbidity subgroup analysis of eAKI for poor outcome page 5](#_Table_2_amount)

# Subgroup Analysis

## Table 1 Admission diagnosis subgroup analysis of eAKI for poor outcome

| Admission diagnosis subgroup |  | N | Crude Relative Risk | Adjusted Relative Risk a |
| --- | --- | --- | --- | --- |
| Sepsis | no eAKI | 234 (52.1%) | Reference | Reference |
|  | Risk | 97 (21.6%) | 1.2 (0.95-1.52; .119) | 1.11 (0.88-1.39; .386) |
|  | Injury | 64 (14.3%) | 1.35 (1.06-1.72; .017) | 1.13 (0.89-1.44; .304) |
|  | Failure | 54 (12.0%) | 1.52 (1.18-1.94; .001) | 1.27 (0.99-1.63; .059) |
|  |  |  |  |  |
| Subarachnoid haemorrhage | no eAKI | 165 (88.7%) | Reference | Reference |
|  | Risk | 18 (9.7%) | 1.37 (0.96-1.98; .085) | 1.22 (0.87-1.70; .246) |
|  | Injury | 3 (1.6%) | 1.37 (0.61-3.11; .444) | 1.44 (0.67-3.09; .348) |
|  | Failure | 0 (0%) | -- | -- |
|  |  |  |  |  |
| Traumatic brain injury | no eAKI | 202 (8.2%) | Reference | Reference |
|  | Risk | 40 (15.9%) | 1.19 (0.63-2.25; .598) | .95 (0.48-1.90; .892) |
|  | Injury | 10 (4.0%) | 1.13 (0.78-1.62; .514) | .87 (0.60-1.24; .437) |
|  | Failure | 0 (0%) | -- | -- |
|  |  |  |  |  |
| Cardiac, non-surgical | no eAKI | 154 (57.9%) | Reference | Reference |
|  | Risk | 65 (24.4%) | 1.18 (0.88-1.58; .264) | 1.15 (0.86-1.53; .347) |
|  | Injury | 35 (13.2%) | 1.44 (1.07-1.94; .015) | 1.25 (0.95-1.66; .112) |
|  | Failure | 12 (4.5%) | 1.46 (0.74-2.86; .268) | 1.35 (0.69-2.64; .372) |
|  |  |  |  |  |
| Other | no eAKI | 833 (65.7%) | Reference | Reference |
|  | Risk | 236 (18.6%) | 1.02 (0.85-1.22; .820) | .96 (0.80-1.15; .649) |
|  | Injury | 141 (11.1%) | 1.34 (1.11-1.62; .002) | 1.16 (0.96-1.39; .127) |
|  | Failure | 57 (4.5%) | 1.73 (1.36-2.21; <.001) | 1.29 (1.01-1.66; .049) |

Results were pooled from 35 imputation datasets, using Rubin’s rule. a, adjusted for age, gender, Charlson comorbidity index, pre-intensive care unit admission hospital length of stay, admission type, acute physiology score (without creatinine), mechanical ventilation in the first 24 hours of admission and confirmed infection in the first 24 hours of admission. Age was transformed into ((age-16)/100)^2, APS was transformed into ((APS-1)/10)^-1+((APS-1)/10); 95%CI, 95% confidence interval; eAKI, early acute kidney injury; ICU, intensive care unit; HRQoL, health related quality of life;

## **Box 1. Subgroup definitions**

| Admission diagnosis groups, based on APACHE admission diagnoses  Sepsis   - NO - Abscess, neurologic - NO - Arthritis, septic - NO - Cellulitis and localized soft tissue infections - NO - Cholangitis - NO - Encephalitis - NO - Endocarditis - NO - GI Perforation/rupture - NO - GI Abscess/cyst - NO - Meningitis - NO - Myositis, viral - NO - Pericarditis - NO - Peritonitis - NO - Pneumonia, bacterial - NO - Pneumonia, fungal - NO - Pneumonia, other - NO - Pneumonia, parasitic (i.e. Pneumocystis pneumonia) - NO - Pneumonia, viral - NO - Renal infection/abscess - NO - Sepsis, cutaneous/soft tissue - NO - Sepsis, GI - NO - Sepsis, other - NO - Sepsis, pulmonary - NO - Sepsis, renal/UTI (including bladder) - NO - Sepsis, unknown - OP - Abscess/infection-cranial, surgery for - OP - Cellulitis and localized soft tissue infections, surgery for - OP - Cholecystectomy/cholangitis, surgery for (gallbladder removal) - OP - Fistula/abscess, surgery for (not inflammatory bowel disease) - OP - GI Perforation/rupture, surgery for - OP - Infection/abscess, other surgery for - OP - Peritonitis, surgery for - OP - Sepsis, cutaneous/soft tissue - OP - Sepsis, GI - OP - Sepsis, gynecologic - OP - Sepsis, other - OP - Sepsis, pulmonary - OP - Sepsis, renal/UTI (including bladder) - OP - Sepsis, unknown - OP - Thoracotomy for thoracic/respiratory infection   Subarachnoid haemorrhage   - NO - Subarachnoid hemorrhage/arteriovenous malformation - NO - Subarachnoid hemorrhage/intracranial aneurysm - OP - Subarachnoid hemorrhage/intracranial aneurysm, surgery for   Traumatic brain injury   - NO - Head (CNS) only trauma - NO - Head/abdomen trauma - NO - Head/chest trauma - NO - Head/extremity trauma - NO - Head/face trauma - NO - Head/multiple trauma - NO - Head/pelvis trauma - NO - Head/spinal trauma - OP - Hematoma, epidural, surgery for - OP - Hematoma, subdural, surgery for - OP - Hemorrhage/hematoma-intracranial, surgery for - OP - Head (CNS) only trauma, surgery for - OP - Head/abdomen trauma, surgery for - OP - Head/chest trauma, surgery for - OP - Head/extremity trauma, surgery for - OP - Head/face trauma, surgery for - OP - Head/multiple trauma, surgery for - OP - Head/pelvis trauma, surgery for - OP - Head/spinal trauma, surgery for   Cardiac, non-surgical   - NO - Angina, stable (asymp or stable pattern of symptoms w/meds) - NO - Angina, unstable (angina interferes w/quality of life or meds are tolerated poorly) - NO - Cardiac arrest (with or without respiratory arrest; for respiratory arrest see Respiratory System) - NO - CHF, congestive heart failure - NO - Infarction, acute myocardial (MI), ANTERIOR - NO - Infarction, acute myocardial (MI), INFEROLATERAL - NO - Infarction, acute myocardial (MI), NON Q Wave - NO - Infarction, acute myocardial (MI), none of the above - NO - Shock, cardiogenic   NO, non-operative; PO, postoperative; UTI, urine tract infection; GI, gastro-intestinal; CNS, central nervous system.  Adapted from Soliman IW, de Lange DW, Peelen LM, Cremer OL, Slooter AJ, Pasma W, Kesecioglu J, van Dijk D: Single-center large-cohort study into quality of life in Dutch intensive care unit subgroups, 1 year after admission, using EuroQoL EQ-6D-3L. J Crit Care. 2015 Feb;30(1):181-6. |
| --- |

## Table 2 amount of comorbidity subgroup analysis of eAKI for poor outcome

| Amount of Charlson comorbidities (not weighted) |  | N | Crude Relative Risk | Adjusted Relative Risk |
| --- | --- | --- | --- | --- |
| 0 | no eAKI | 551 (72.9%) | Reference | Reference |
|  | Risk | 130 (17.2%) | 1.21 (0.93-1.57; .149) | 0.99 (0.77-1.27; .916) |
|  | Injury | 48 (6.3%) | 1.5 (1.07-2.09; .018) | 1.14 (0.81-1.61; .446) |
|  | Failure | 27 (3.6%) | 1.68 (1.07-2.63; .024) | 1.18 (0.71-1.96; .515) |
|  |  |  |  |  |
| 1 | no eAKI | 511 (69.4%) | Reference | Reference |
|  | Risk | 139 (18.9%) | 1.17 (0.96-1.41; .113) | 1.11 (0.91-1.34; .301) |
|  | Injury | 64 (8.7%) | 1.39 (1.11-1.74; .004) | 1.18 (0.94-1.48; .152) |
|  | Failure | 22 (3%) | 1.74 (1.37-2.2; <.001) | 1.35 (1.05-1.74; .020) |
|  |  |  |  |  |
| 2 | no eAKI | 298 (60.9%) | Reference | Reference |
|  | Risk | 90 (18.4%) | 0.94 (0.72-1.23; .649) | 0.87 (0.68-1.12; .282) |
|  | Injury | 67 (13.7%) | 1.22 (0.96-1.55; .098) | 1.1 (0.87-1.4; .417) |
|  | Failure | 34 (7%) | 1.44 (1.06-1.94; .019) | 1.22 (0.89-1.67; .206) |
|  |  |  |  |  |
| 3 or more | no eAKI | 228 (51.9%) | Reference | Reference |
|  | Risk | 97 (22.1%) | 1.01 (0.8-1.29; .908) | 0.94 (0.74-1.19; .609) |
|  | Injury | 74 (16.9%) | 1.09 (0.86-1.39; .471) | 1.06 (0.85-1.33; .592) |
|  | Failure | 40 (9.1%) | 1.41 (1.07-1.87; .017) | 1.17 (0.87-1.57; .290) |

Results were pooled from 35 imputation datasets, using Rubin’s rule. a, adjusted for age, gender, Charlson comorbidity index, pre-intensive care unit admission hospital length of stay, admission type, acute physiology score (without creatinine), mechanical ventilation in the first 24 hours of admission and confirmed infection in the first 24 hours of admission. Age was transformed into ((age-16)/100)^2, APS was transformed into ((APS-1)/10)^-1+((APS-1)/10); 95%CI, 95% confidence interval; eAKI, early acute kidney injury; ICU, intensive care unit; HRQoL, health related quality of life;
